# Age and Clinical Outcomes of Immune Checkpoint Inhibitor Toxicities in Portugal: A Decade of Pharmacovigilance

**DOI:** 10.3390/cancers18010076

**Published:** 2025-12-25

**Authors:** Tiago Pina-Cabral, José Pereira, João Paulo-Fernandes, Márcia Silva, Mário Fontes-Sousa, Mariana Anacleto, Soraia Lobo-Martins, Ana Mirco, Helena Miranda, Ana Martins, Patrícia Cavaco

**Affiliations:** 1 Unidade Local de Saúde de Lisboa Ocidental, 1449-005 Lisbon, Portugalpcavaco@ulslo.min-saude.pt (P.C.); 2 Unidade de Farmacovigilancia, Direção de Gestão do Risco de Medicamentos, INFARMED—Autoridade Nacional do Medicamento e Produtos de Saúde, I.P., 1749-004 Lisbon, Portugal; 3 CUF Tejo Hospital, 1350-352 Lisbon, Portugal; 4 Academic Trials Promoting Team, Institut Jules Bordet, Hôpital Universitaire de Bruxelles (H.U.B), Université libre de Bruxelles (ULB), 1070 Brussels, Belgium

**Keywords:** immune checkpoint inhibitors, immune-related adverse events, pharmacovigilance, real-world evidence, elderly, CTLA-4, Portugal

## Abstract

**Background**: Real-world safety profiles of immune checkpoint inhibitors (ICIs) in older adults remain insufficiently characterized. Although ICIs are widely used across tumor types, older patients, particularly those with frailty, multimorbidity, or polypharmacy, are consistently under-represented in clinical trials, limiting the external validity of trial-derived toxicity estimates. Robust real-world data are therefore essential to clarify the incidence, seriousness, and age-related patterns of immune-related adverse events (irAEs) in routine practice. **Methods**: This is a nationwide retrospective study of spontaneous ICI-related ADRs reported in INFARMED’s Portal RAM (2011–2024). We evaluated the frequency, seriousness, fatality, and organ-specific patterns of ICI-related adverse drug reactions (ADRs) reported to the Portuguese National Pharmacovigilance System. The analytic unit was the ADR case. Endpoints included seriousness (primary), fatality, hospitalization, time-to-onset, and System Organ Class. Multivariable logistic regression adjusted for age, sex, regimen, tumor type, polypharmacy, and calendar period; sensitivity analyses using first ADR per patient were concordant. **Results**: We identified 2300 eligible ICI-related ADRs (corresponding to 925 patients). Median age at the time of ADR was 65 years (IQR not reported); 33.7% occurred in adults aged ≥70 years, and 62.8% of reports involved male patients. PD-1 inhibitors accounted for 77.5% of ADRs, and monotherapy for 72.9%. Overall, 85.8% of ADRs were classified as serious; 17.9% led to hospitalization and 19.1% were fatal. Serious-event reporting was similar in older and younger adults (≥70 vs. <70 years: 84.5% vs. 86.5%, *p* = 0.22), and the proportion explicitly labeled immune-related did not differ (9.3% vs. 8.7%, *p* = 0.56). In contrast, fatal outcomes were significantly more common in older adults (25.3% vs. 16.0%; *p* < 0.001). Age was associated with distinct organ-specific patterns. Adults ≥ 70 years had higher odds of nervous system disorders (aOR 1.75, 95% CI 1.23–2.48) and immune system disorders (aOR 1.42, 95% CI 1.02–1.98), but lower odds of hepatobiliary (aOR 0.52, 95% CI 0.36–0.76; *p* = 0.001) and blood/lymphatic disorders (aOR 0.50, 95% CI 0.32–0.79). In multivariable models, age ≥ 70 years did not predict seriousness (aOR 0.98, 95% CI 0.76–1.27), whereas combination therapy remained independently associated with increased seriousness (aOR 1.57, 95% CI 1.13–2.18). Conversely, age ≥ 70 years independently predicted fatal outcomes (aOR 1.66, 95% CI 1.31–2.09). Later calendar periods (2017–2024) were associated with substantially lower fatality (aOR 0.16; 95% CI 0.10–0.27). CTLA-4-containing regimens demonstrated a tendency toward higher fatality (aOR 1.50; 95% CI 0.94–2.37). **Conclusions**: Chronological age does not seem to increase the likelihood of reporting a serious ICI-related ADR, but, once toxicity occurs, older adults experience higher fatality rates. Age-related phenotypic differences and regimen-specific risks highlight the need for early recognition systems and tailored toxicity management in older populations.

## Simple Summary

Immune checkpoint inhibitors (ICIs) are widely used across many cancer types, yet their real-world safety in older adults is not well studied. Using a decade of national pharmacovigilance data from Portugal, we examined adverse drug reactions (ADRs) reported in patients treated with ICIs, focusing on whether age impacts the seriousness or pattern of toxicities. We found that serious ADRs were not more frequently reported in older adults than younger adults; however, when severe toxicity occurred, fatal outcomes were more common in patients aged 70 years or older. The types of organ systems affected also differed by age, with nervous and immune system events more frequently reported in older adults and hepatic or hematologic events more commonly in younger patients. Treatment regimen, particularly combination therapy, also influenced the likelihood of being classified as serious. These findings highlight the importance of early recognition and tailored management of ICI-related toxicities in older patients, rather than excluding them from this treatment based on age alone. Highlights/Key messages: Chronological age did not increase the odds of serious ICI ADRs; however, patients aged ≥70 years had higher fatality once toxicity occurred (aOR 1.66, 95% CI 1.31–2.09). Toxicity phenotype differed by age: more nervous/immune system events in patients aged ≥70 years, and more hepatobiliary/hematologic events in patients aged <70 years. Combination regimens involving anti-PD-1/PD-L1 plus anti-CTLA-4 were independently associated with a higher likelihood of being classified as a serious adverse drug reaction (aOR 1.57, 95% CI 1.13–2.18). However, in adjusted analyses, combination therapy was not associated with increased odds of fatal outcome. In contrast, CTLA-4–containing regimens showed the highest crude proportions of fatal reports in the cohort, reflecting their overall more severe toxicity profile.

## 1. Introduction

The advent of immune checkpoint inhibitors (ICIs) has redefined the therapeutic landscape of oncology, yielding survival benefits across diverse malignancies [1,2,3,4,5]. By targeting key regulatory pathways, such as cytotoxic T-lymphocyte–associated antigen 4 (CTLA-4), programmed death 1 (PD-1), programmed death ligand 1 (PD-L1), and more recently, lymphocyte-activation gene 3 (LAG-3), ICIs enhance the intrinsic ability of the immune system to recognize and eliminate malignant cells. They have been approved for use in both (neo)adjuvant and metastatic settings, offering unprecedented prospects for patients with historically poor prognoses. Since the approval of ipilimumab (anti-CTLA-4) in Europe in 2011, the therapeutic arsenal has steadily expanded. Notably, subsequent approvals of nivolumab and pembrolizumab (anti-PD-1) in 2015, atezolizumab and avelumab (anti-PD-L1) in 2017, durvalumab (anti-PD-L1) in 2018, and more recently, cemiplimab (anti-PD-1) along with novel combination regimens such as nivolumab/relatlimab (anti-PD-1/LAG-3) and durvalumab/tremelimumab (anti-PD-L1/CTLA-4), have markedly broadened treatment options.

Unlike traditional cytotoxic therapies, ICIs unleash the full potential of the immune system, offering substantial therapeutic benefit against malignancies. However, this enhanced immune activity may inadvertently result in non-tumor immune-mediated damage, producing a unique spectrum of toxicities termed immune-related adverse events (irAEs) that can affect virtually any organ system [6,7,8,9,10,11,12,13]. Of these, the most commonly affected systems include the skin, lungs, colon, and endocrine glands [4,9,13,14,15]. The clinical spectrum of irAEs ranges from mild dermatologic eruptions or thyroid dysfunction to severe and potentially fatal conditions, such as pneumonitis, myocarditis, colitis, and neuroinflammatory syndromes. In clinical trials, grade 3 or higher, irAEs occur in approximately 15–20% of patients treated with anti-PD-1/PD-L1 monotherapy, whereas this rate escalates to over 50% within combination regimens involving anti-CTLA-4 [16,17]. Early recognition and prompt management, typically with corticosteroids or other immunosuppressive agents, are critical for mitigating morbidity and mortality, although these interventions can potentially compromise antitumor efficacy and influence long-term outcomes [15,18]. Beyond direct toxicities, emerging data reveal that concomitant medications, particularly antibiotics, may disrupt the gut microbiome and thereby diminish ICI efficacy [19]. Electrolyte imbalances, notably among lung cancer patients, further complicate clinical management, underscoring the necessity for proactive monitoring during immunotherapy.

Despite the robustness of clinical trial data, translating these findings into routine clinical practice remains challenging, largely due to disparities in patient demographics and comorbidities. Real-world data in ICI toxicity are relatively limited, since clinical trial populations are often highly selective and may under-represent older adults and those with comorbidities. Notably, patients over 80 years of age comprise approximately 16% of cancer cases but only constitute approximately less than 4% of trial participants [20,21,22], raising concerns regarding the generalization of trial-derived safety and efficacy data to real-world geriatric populations. Despite the high burden of comorbidity and polypharmacy in older adults, the more favorable safety profile of ICIs compared with cytotoxic chemotherapy has enabled their increasing adoption in this age group [20,23,24,25]. On the other hand, *immunosenescence*, the age-related remodeling of the immune system, does not simply attenuate immune responses but can paradoxically promote dysregulated immunity. Age-associated thymic involution, expansion of autoreactive memory T-cell clones, and chronic low-grade inflammation (“inflammaging”) may increase susceptibility to certain immune-mediated toxicities rather than protect against them [26,27,28]. In this context, the frailty, multimorbidity and reduced physiological reserve characteristic of older adults can further amplify the clinical impact of irAEs, contributing to the higher case-fatality observed in this population [26,27]. The existing literature remains inconclusive: while some studies have found no significant difference in overall irAE frequency between older and younger adults, whereas others, including pharmacovigilance analyses, suggest older patients may have higher reporting rates of specific irAEs [6,22,23]. A large cohort study of 19,177 patients aged ≥ 75 years reported no significant difference in irAEs incidence compared to younger cohorts, a finding corroborated in an analysis of 928 patients aged ≥80 years, where 41.3% experienced at least one irAE with consistent patterns across age groups [25]. Nonetheless, geriatric syndromes such as frailty, polypharmacy, and multimorbidity have emerged as critical modifiers of risk, with frail patients demonstrating heightened susceptibility to irAEs, thus emphasizing the need for comprehensive geriatric assessments to inform clinical decision-making [25,29,30]. The toxicity profile also seems to shift with age; for example, some studies indicate that endocrine irAEs (such as thyroiditis/hypophysitis) appear more commonly in younger patients, while dermatologic irAEs occur more frequently in patients ≥ 75 years old [31]. Additionally, elderly patients might have higher mortality rates and longer hospital stays when severe irAEs occur [13,14,15,22]. It is largely unknown whether elderly patients experience different incidences or patterns of irAEs compared to younger patients. Given these uncertainties, real-world data focusing on older adults are crucial to better characterize toxicity risks and inform clinical decision-making in this growing population.

Recognizing the limitations of clinical trial data, pharmacovigilance systems capture real-world safety profiles in heterogeneous populations. In Portugal, adverse drug reactions are reported to the National Pharmacovigilance System (Portal RAM), which collects spontaneous notifications from healthcare professionals and patients. These data enable the early detection of safety signals, characterization of toxicity in underrepresented groups, and identification of risk factors for severe irAEs, particularly in older or comorbid patients. Despite international evidence supporting real-world analyses, no systematic evaluation of ICI-related events has been conducted in Portugal to date.

The *Immunotherapy Monitoring in Portugal: Age and Clinical Toxicity Profiling (The IMPACT Study)* was designed to assess the frequency, seriousness, and organ-specific distribution of irAEs reported to INFARMED, with an emphasis on age and clinical determinants. By including all European Commission authorized ICIs, the IMPACT Study aimed not only to supplement evidence from clinical trials but also to promote safer, more individualized use of immunotherapy among vulnerable patient populations in routine practice.

## 2. Materials and Methods

This study was designed as a national, retrospective observational cohort of ICI reported adverse events, utilizing Portugal’s pharmacovigilance database. All spontaneous adverse drug reaction (ADR) reports submitted to INFARMED’s “*Portal RAM*” system from 1 January 2011, through 31 December 2024, were screened. In Portugal, ADRs are reported to the National Pharmacovigilance System by healthcare professionals, pharmaceutical companies, and patients/caregivers, through the online Portal RAM. Reports are anonymized by INFARMED before release for research purposes, ensuring that no directly identifiable information is accessible to investigators. The system does not retain a variable indicating whether a notification originated from a clinical study or routine care; therefore, all ICSRs in this dataset were operationally considered spontaneous reports, consistent with the absence of mandatory prospective reporting in Portal RAM during the study period. To minimize duplication, INFARMED applies internal quality control procedures before database extraction; however, because the dataset does not include patient identifiers beyond age and sex, we could not independently verify duplicate submissions. Potential clustering due to multiple ADRs per individual was addressed analytically by conducting sensitivity analyses restricted to the first ADR per patient, which yielded results concordant with the primary case-level analyses. Reports were eligible if they involved at least one EMA-approved ICI, such as anti-PD-1 (e.g., nivolumab, pembrolizumab and cemiplimab), anti-PD-L1 (atezolizumab, avelumab and durvalumab), anti-CTLA-4 (ipilimumab and tremelimumab), or other agents approved in Europe. Each individual case safety report (ICSR) corresponded to a single patient who experienced one or more reported ADR associated with ICI treatment. We excluded entries that did not represent a safety related adverse reaction (e.g., reports of medication error, lack of efficacy, or unrelated medical issues) and those with critical missing data. After applying the inclusion criteria, a total of 2300 eligible ICI-related ADR cases were identified, corresponding to 925 unique patients, as some individuals contributed more than one ADR report.

### 2.1. Data Sources and Variables

For each ADR report, we extracted patient demographics, such as age at ADR, sex, cancer type/indication, ICI drug(s) administered, and concomitant medications. Age was treated as a continuous variable (for summary statistics) and dichotomized into two groups (<70 vs. ≥70 years) for the primary subgroup analyses. Tumor types were categorized into major groups (non-small cell lung cancer (NSCLC), melanoma, renal cell carcinoma, breast cancer, and “other” malignancies) based on the indications listed. The treatment regimen was classified as monotherapy, a single ICI agent, or combination therapy, defined as an ICI administered in combination with at least one other anticancer agent (such as another ICI or chemotherapy). Additionally, we specifically flagged CTLA-4-containing regimens to examine their risk profiles. The number of concomitant medications at the time of the ADR was counted and stratified into three categories: ≤2, 3–5, and >5 concurrent noncancer treatment drugs. Each ADR was coded using MedDRA terminology. We collated the involved primary System Organ Classes (SOCs) to characterize the affected organ systems. Events were further labeled as irAEs when the notifier indicated an immune-mediated mechanism. The ‘immune-related’ label reflects the notifier’s clinical judgement rather than an adjudicated or algorithm-based irAE classification; all ADRs were first coded as specific MedDRA Preferred Terms and then mapped to System Organ Classes, but a given clinical entity (e.g., thyroiditis, hepatitis, colitis) may or may not have been explicitly designated as immune-mediated by the reporter, leading to under-capture of irAEs within this variable. The outcomes of each ADR case were recorded in terms of seriousness and specific serious outcome criteria. Seriousness was defined according to standard pharmacovigilance criteria (death, life-threatening event, hospitalization, disability, or other medically important condition). For the primary endpoint of this study, we operationally defined severe irAEs as any ADR meeting the serious criteria, focusing on cases involving hospitalization, life-threatening conditions, or fatal outcomes. Accordingly, fatal outcomes and hospitalizations were analyzed as key subcategories of seriousness of AE. Reporter characteristics (healthcare professional vs. patient) and timing variables were also collected. The calendar year of ICI start and of ADR occurrence were noted, and when available, the time-to-onset of the toxicity was calculated (interval from ICI initiation to ADR onset). All data were de-identified; each case was indexed only by a coded ID with no personal identifiers, in compliance with national data protection regulations. The analytic unit was the ADR case (i.e., a unique report fulfilling the inclusion criteria). Multiple ADRs can originate from the same patient. For transparency, we conducted: (i) a primary case-level analysis; and (ii) a sensitivity analysis restricted to the first eligible ADR per patient (de-duplicated patient-level dataset). Analyses were performed at the case level (ADR reports). Because some patients contributed >1 ADR, we performed a prespecified sensitivity analysis restricted to each patient’s first eligible ADR; the results were concordant. Cluster-robust standard errors by patient were not universally feasible due to incomplete linkage; therefore, we present conservative case-level CIs and first-event results. Given the spontaneous-reporting nature of the INFARMED pharmacovigilance system, all events coded by the notifier as “death” were retained without re-adjudication of causality, in accordance with standard pharmacovigilance procedure. As such, these figures represent the frequency of ADR reports labelled as fatal, not the incidence or prevalence of treatment-related mortality, and are subject to well-recognized severity-reporting inflation. Each fatal entry was counted once per ICSR, and internal data-cleaning procedures removed within-report duplicate entries; however, misclassification between ADR-related and co-occurring deaths cannot be fully excluded in spontaneous notification systems. Although the dataset includes whether death was reported as an outcome, the Portal RAM system does not record a structured date of death; therefore, the interval between ADR onset and fatal outcome could not be computed, and time-to-death analyses were not feasible.

### 2.2. Ethical Considerations

The study was approved by the Unidade Local de Saúde Lisboa Ocidental Ethics Committee, with authorization for use of anonymized INFARMED pharmacovigilance data; informed consent was not required under national regulations and the Declaration of Helsinki.

### 2.3. Statistical Analysis

All statistical analyses were performed using a contemporary software environment (SPSS Statistics version 26) on the final dataset of 2300 ADR cases. We first performed descriptive analyses to summarize the cohort characteristics. Continuous variables are reported as means with standard deviations or medians with interquartile ranges. Categorical variables are presented as frequencies and percentages of the total. The primary comparison of interest was between the younger (<70 years) and older (≥70 years) patient groups. We used chi-square tests (or Fisher’s exact test when expected cell counts were <5) to compare proportions between these age strata for categorical endpoints. Key comparisons included the proportion of cases classified as serious versus non-serious, the occurrence of fatal outcomes, hospitalizations, and the distribution of organ-specific irAEs in older versus younger patients. For continuous or ordinal outcomes (e.g., time-to-onset of an ADR), we assessed group differences using non-parametric tests (Mann–Whitney U) given the skewed distributions; for instance, the time-to-onset variable was not normally distributed. A two-sided α-level of 0.05 was used to determine statistical significance for all hypothesis tests. We evaluated multiple secondary comparisons across the other subgroups. These included analyses by sex (male vs. female), tumor group, treatment type, regimen (monotherapy vs. combination), CTLA-4 use, polypharmacy category, and calendar period (early vs. late cohort). For each of these, contingency tables were constructed for outcomes such as serious vs. non-serious rates, fatality, hospitalization, and immune-related event frequency, and tested using the chi-square test. Given the number of comparisons, we interpreted *p*-values with caution; the findings were highlighted as exploratory. No formal adjustment for multiple testing was applied because the analyses were largely descriptive; however, we emphasized consistent patterns over isolated *p*-value significance.

To identify independent predictors of severe toxicity, we performed multivariable logistic regression modeling of the odds of a report being serious versus non-serious. Candidate predictors were chosen a priori based on clinical interest and the univariate results. The final model included age group (≥70 vs. <70), sex, treatment regimen (combination vs. monotherapy), CTLA-4 regimen (whether the ICI regimen included a CTLA-4 inhibitor), polypharmacy (treated as an ordinal variable by category), tumor type (with dummy indicators for melanoma, NSCLC, renal cell carcinoma, and breast cancer, using all other tumors as the reference category), and calendar period (2017–2024 vs. 2011–2016). These variables were entered simultaneously into the logistic model to adjust for potential confounding factors and to estimate each factor’s independent association with severe irAE occurrence. The results are reported as odds ratios (OR) with 95% confidence intervals and *p*-values for each covariate. Model fit was assessed using the pseudo R-squared and Hosmer–Lemeshow goodness-of-fit tests. In a second multivariable analysis, we examined predictors of fatal outcomes using logistic regression on a subset of serious cases (outcome: death vs. non-fatal outcome), incorporating the same covariates. Similarly, a model for hospitalization was explored. These additional models were considered to be exploratory. We checked for multicollinearity among predictors (for example, CTLA-4 use is largely encompassed by combination therapy status) and found acceptable variance inflation factors. No interaction terms were included in the final models, given the limited sample size for higher-order interactions; however, we specifically examined whether age might modify the effect of the regimen in stratified analyses. The results of the subgroup analyses are described to corroborate the main findings. All statistical tests were performed using two-tailed tests. Analyses and data visualization followed the ESMO recommendations for real-world evidence reporting, and all figures and tables were generated directly from the analyzed dataset.

### 2.4. Missing Data

We quantified the variable-level missingness prior to the analysis. Time-to-onset was available for 542/2300 ADR cases (23.6%); thus, 76.4% were missing. Missingness was low for core covariates used in the multivariable models (tumor type, ICI class/regimen, age group, sex, calendar period, and polypharmacy), and these records were excluded from the relevant models. Therefore, all regression analyses were conducted as complete-case analyses, and no imputation was performed. Descriptive percentages were reported using non-missing denominators and footnoted accordingly. Sensitivity analyses excluding the “unknown/not reported” categories (where present) yielded estimates consistent with the primary results.

## 3. Results

**Figure 1:**
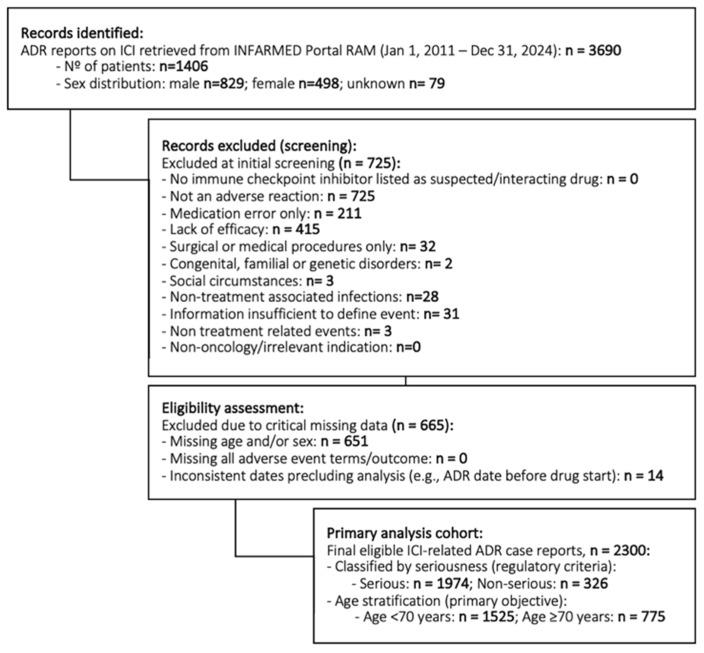
Flow diagram of the IMPACT study cohort derived from the Portuguese National Pharmacovigilance System (Portal RAM/INFARMED). The diagram summarizes the number of adverse drug reactions (ADRs) screened, exclusions applied, and the final cohort included for analysis (1406 patients/3690 ADRs to the analytic dataset (925 patients/2300 ADRs).

Of the 3690 ADRs screened, from 1406 patients, 2300 unique ICI-related ADRs met the inclusion criteria, corresponding to 925 patients (Figure 1). The patients’ ages ranged from 20 to 93 years, with a median age of 65. One-third of the ADR cases (33.7%, *n* = 775) involved older adults (≥70 years), while the remaining 66.3% (*n* = 1525) were younger than 70 years. Men accounted for most of the reports (62.8%). The most common cancer type was lung cancer (31.3%), followed by melanoma (15.5%) and breast cancer (8.1%), respectively. Other malignancies accounted for 45% of the reports (including genitourinary, gastrointestinal, head-neck cancers, and others not enumerated). Regarding ICI treatments, PD-1 inhibitors were implicated in 77.5% of the reports (*n* = 1783), PD-L1 inhibitors in 15.2%, and CTLA-4 inhibitors in 4.9%, either alone or in combination (2.4% involved dual ICI combinations, such as anti–PD-1 plus anti–CTLA-4). In 72.9% of cases, ICI was administered as monotherapy, whereas 27.1% were combination regimens. Regarding the burden of comorbid pharmacology, over one-fifth of patients (11.2%) were on >5 concomitant medications at the time of the ADR, with an additional 11.8% on 3–5 other drugs, and 77.0% on 0–2 concomitant medications (Table 1).

The majority of ICI-related ADRs were categorized as “serious”: 1974 out of 2300 reports (85.8%) met the regulatory definition of a serious ADR, and 326 (14.2%) were classified as non-serious events. Among serious cases, the specific outcomes varied according to clinical seriousness. Fatal outcomes were reported in 19.1% of all cases (*n* = 440), corresponding to a 1:5 ratio of deaths associated with ICI-induced ADRs. Similarly, hospitalizations were reported in 17.9% of the cases (*n* = 412). “*Important medical event*” was the most frequently selected seriousness criterion (53.7%). A breakdown of cases by CTCAE-equivalent grade extrapolation illustrates this distribution: 19.1% of cases corresponded to Grade 5 (death), approximately 6.7% to life-threatening (Grade 4) events, and 6.3% to serious events leading to hospital stay or disability (Grade 3), while the remaining serious reports were categorized as medically important significant events not immediately life-threatening, 53.7% (Grade 2). Only 14.2% of reports were truly mild (non-serious, roughly corresponding to CTCAE grade 1). Detailed cross-tabulations of seriousness, immune-related labeling, hospitalization, and CTCAE-equivalent grades by age group are provided in Supplementary Table S1, with graphical grade distributions shown in Supplementary Figure S1. irAEs were further differentiated in immune-mediated, those considered triggered by immune activation from checkpoint blockade, versus non-immune-mediated events; overall, 9.0% of the reports (*n* = 207) were judged to represent immune-mediated toxicities (such as autoimmune pneumonitis, colitis, hepatitis and endocrinopathies), whereas the vast majority (91.0%) were classified as “*other*” events that are either not typical irAEs or not clearly immune in origin (for example, infusion reactions, infections, diarrhea, ataxia, or other complications). The median time from treatment initiation to ADR onset was 30 days (0–90 days) in the subset of reports with available timing data (*n* = 542); however, a large fraction of reports had an onset within the first few days of therapy, resulting in an overall median of 0 days in both age groups.

**Figure 2:**
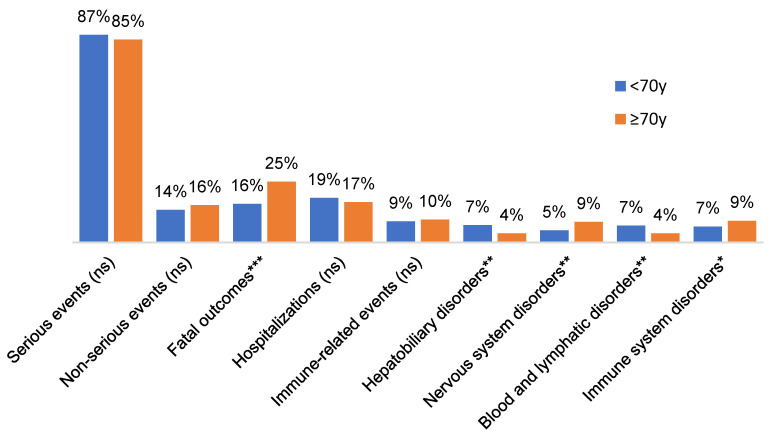
Age-Stratified Distribution of Adverse Drug Reaction Outcomes and Selected Organ-System Involvement. Distribution of adverse drug reaction (ADR) outcomes and selected MedDRA System Organ Classes (SOC) in patients treated with immune checkpoint inhibitors, stratified by age group (<70 vs. ≥70 years). Bars represent proportions of serious events, non-serious events, fatal outcomes, hospitalizations, immune-related events, and selected SOC categories. Comparisons between age groups were evaluated using chi-square tests; significant differences are indicated by asterisks. Full numerical values for these categories are provided in Supplementary Table S1. *, *p* < 0.05; **, *p* < 0.01; ***, *p* < 0.001; ns, not significant.

**Figure 3:**
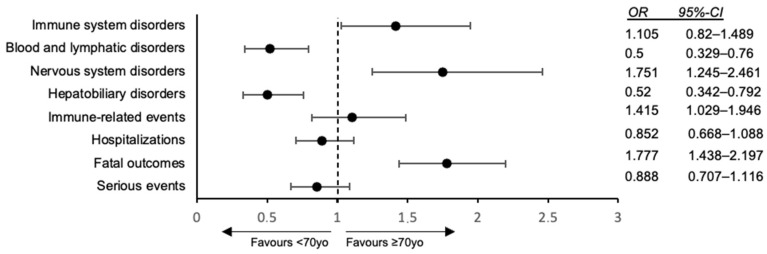
Age-related differences in immune checkpoint inhibitor (ICI) toxicity: odds ratios (≥70 vs. <70 years) with 95% CIs. Univariable odds ratios (ORs) with 95% confidence intervals (CIs) comparing patients aged ≥70 years with those <70 years for selected adverse drug reaction (ADR) outcomes and organ-system categories. The dotted vertical line indicates OR = 1 (no age effect).

Age-stratified comparisons of outcomes between patients ≥ 70 years and those younger revealed that elderly patients did not experience a higher overall frequency of serious ADRs: the proportion of reports classified as serious was 84.5% in the ≥70 group (655/775) versus 86.5% in those <70 (1319/1525), a difference that was not statistically significant (*p* = 0.22) (Figure 2). However, fatal irAEs were markedly more common in elderly patients than in younger patients. Among the ≥70 group, 25.3% of cases were fatal, compared to 16.0% in <70 adults. This 1.6-fold increase in fatal outcome frequency in the elderly was highly significant (*p* < 0.001) and corresponded to an excess risk of death attributable to irAEs in older patients. In absolute terms, 196 of 775 reports in the ≥70 cohort involved patient death versus 244 of 1525 reports in younger patients. In contrast, the rate of hospitalization did not differ appreciably by age: 16.6% of older patients had an ADR leading to hospital admission vs. 18.5% of younger patients (*p* = 0.21), indicating a similar likelihood of requiring inpatient care for managing toxicity. The fraction of events deemed immune-related (9% in ≥70 vs. 8.7% in <70) was also similar (*p* = 0.56). Thus, aside from mortality, the overall seriousness and broad categories of events were comparable between the age groups. Despite comparable aggregate serious-event rates, the organ-system distribution differed modestly by age. Older adults had a higher representation of events coded under *Immune system disorders* (9.0% vs. 6.6%, *p* = 0.039), largely driven by immune-mediated endocrinopathies (e.g., thyroiditis), immune-mediated skin disorders, and a relative enrichment of myocarditis, patterns consistent with the age-related shift observed in prior studies. Conversely, younger patients contributed proportionally more cases of immune-mediated colitis and hepatitis They also had significantly more nervous system disorders reported (8.5% vs. 5.0%, *p* = 0.002), suggesting neurologic irAEs (such as immune-related neuropathies or encephalitis) were relatively more frequent in older individuals. In contrast, younger patients showed higher rates of hepatic and biliary ADR (7.2% of <70 reports vs. only 3.7% in ≥70, *p* = 0.001) and more blood or lymphatic disorders (7.0% vs. 3.7%, *p* = 0.003) (Figure 3). There was no significant age difference in several other common organ categories: for example, endocrine disorders were numerically more frequent in older patients (11.1% vs. 8.7% in <70), but this did not reach significance (*p* = 0.07); skin-related irAEs occurred in 9% of cases in both groups (*p* = 0.31). Gastrointestinal (colitis) and respiratory (pneumonitis) event rates were also similar by age (8% in both groups) (Table 2). The median time-to-onset of toxicity did not differ by age: both groups had a median of 0 days from ICI start to event (with an identical proportion of events occurring on day 0, likely infusion reactions or acute events). Considering the non-zero events, the distribution of onset timing was broad and showed no clear shift between age groups (*p* = 0.65 for difference in time-to-onset). A global overview of age-related organ-system involvement across all MedDRA System Organ Classes is shown in Supplementary Figure S2. Age-related shifts in MedDRA System Organ Classes are presented in Supplementary Figure S3, and the most frequently reported adverse events and immune-related events by age group are summarized in Supplementary Table S2. Graphical representations of these age-stratified adverse event patterns are provided in Supplementary Figure S4.

In addition to the age-stratified analyses, we performed exploratory evaluations by sex, tumor type, treatment regimen, polypharmacy, temporal trend and reporter type. Given their descriptive nature, the full cross-tabs and model outputs are provided in Supplementary Materials.

Overall safety outcomes were comparable by sex, with similar rates of serious events (*p* = 0.89) and fatal outcomes (19.8% in males vs. 18.0% in females, *p* = 0.31). Men, however, were more frequently hospitalized (*p* = 0.002) and showed a non-significant trend toward more immune-related events (9.8% vs. 7.7%, *p* = 0.11). Sex-specific patterns emerged, with women reporting more endocrine (11.7% vs. 8.4%, *p* = 0.001) and hematologic disorders, while men had higher frequencies of respiratory and vascular toxicities (1.9% vs. 0.6%; *p* = 0.014); onset timing did not differ. Toxicity patterns varied by tumor type. Melanoma cases had the highest burden, with 89.3% serious events, 36.5% hospitalizations, and 29.8% fatality, while NSCLC (*n* = 719) showed lower seriousness rate (81.6% serious, 14.9% fatal) but the highest frequency of immune-related events (12.9%). Renal cell carcinoma (21.1% fatal, only 2% hospitalized) and breast cancer (93% serious but only 2.7% fatal and no hospitalizations) illustrated marked heterogeneity, with breast cancer patients experiencing the most favorable outcomes. Combination regimens were associated with a higher proportion of serious events than monotherapy (91.0% vs. 84.3%, *p* < 0.001), but paradoxically with lower fatality (13.6% vs. 21.2%, *p* < 0.001) and hospitalization rates (7.5% vs. 21.8%, *p* < 0.001), as well as fewer immune-mediated events (6.1% vs. 10.1%, *p* = 0.004). In contrast, CTLA-4–containing regimens (*n* = 168, predominantly ipilimumab-based) carried a markedly worse profile: although the proportion of serious ADRs was similar to non-CTLA-4 (89.9%), fatality reached 40.5% versus 17.4% (*p* < 0.001) and hospitalizations 43.5% versus 15.9% (*p* < 0.001). Immune-mediated events were also more frequent with CTLA-4 (14.3% vs. 8.6%, *p* = 0.012), despite these regimens being more often used in younger (34% <70 vs. 24% ≥70) and male (61%) patients. Polypharmacy influenced outcomes, as patients on 3–5 concomitant drugs had the highest hospitalization rate (28.7% vs. 16.7% with ≤2 drugs; *p* < 0.001) but the lowest fatality (11.0% vs. 20% in other groups; *p* < 0.001). In contrast, those on >5 medications experienced the highest mortality (22.9%, above the 19.8% baseline; *p* < 0.001) despite a lower overall rate of serious events (80.6%). ADR seriousness reporting declined over time: serious events (91.7% to 85.3%), hospitalizations (32.0% to 16.7%), and fatal outcomes (58.6% to 15.8%) were all significantly less frequent in 2017–2024 compared to 2011–2016. In contrast, the proportion of immune-related events increased (9.5% vs. 3.3%), reflecting improved recognition and reporting. The seriousness of ADRs was consistent across reporter types (*p* = 0.31), with healthcare professionals submitting the majority and patient reports representing 3.5% of cases. Notably, patient-reported events were not less severe, and over half involved fatal outcomes, though absolute numbers were small.

**Figure 4:**
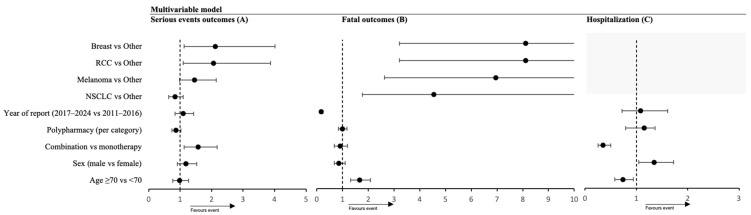
Multivariable logistic regression models for determinants of serious events, fatal outcomes, and hospitalization in patients experiencing ICI-related adverse events. Adjusted odds ratios (ORs) with 95% confidence intervals (CIs) from multivariable logistic regression models evaluating determinants of (**A**) serious adverse drug reactions (ADRs), (**B**) fatal outcomes, and (**C**) hospitalization in patients treated with immune checkpoint inhibitors. Covariates included age group, sex, tumor type, treatment regimen, CTLA-4 use, polypharmacy category, and calendar period. The vertical reference line represents OR = 1. Full regression tables are provided in the Supplementary Materials.

To disentangle the contributions of various factors to severe toxicity, we built a multivariate logistic regression model for the outcome of serious vs. non-serious ADR. In this adjusted analysis, age ≥ 70 was not an independent predictor of severe irAE occurrence (OR = 0.98, 95% CI 0.76–1.27, *p* = 0.89), confirming that older patients, after accounting for other variables, were not more likely than younger patients to experience a reportable event meeting seriousness criteria (Figure 4). Sex likewise had no significant effect on seriousness (male vs. female OR = 1.18, 95% CI 0.91–1.53, *p* = 0.20). In contrast, combination immunotherapy had an adjusted OR of 1.57 (95% CI 1.13–2.18, *p* = 0.007) for producing a serious ADR compared to monotherapy. Combination therapy conferred 57% higher odds of a severe reaction. Neither polypharmacy (OR = 0.87 per category, 95% CI 0.73–1.03, *p* = 0.11) nor calendar period (2017–2024 vs. 2011–2016; OR = 1.09, 95% CI 0.83–1.42, *p* = 0.54) were significant predictors of a serious events. Tumor type was an independent predictor of seriousness: renal cell carcinoma (OR = 2.06, 95% CI 1.09–3.87, *p* = 0.025) and breast cancer (OR = 2.12, 95% CI 1.06–4.02, *p* = 0.021) both showed higher odds of severe ADRs compared with “other” tumors. Melanoma showed a non-significant trend toward increased seriousness (OR = 1.45, *p* = 0.06), while NSCLC did not differ significantly (OR = 0.84, *p* = 0.20). Using breast cancer as the reference confirmed these findings, with lung cancer showing significantly lower odds of seriousness and melanoma marginally lower, underscoring the unexpectedly high seriousness classification in breast cancer despite low fatality. The model’s pseudo-R^2^ was 0.02, indicating that most variability in ADR seriousness remained unexplained, likely reflecting unmeasured toxicity patterns or reporting practices.

In the multivariable model for fatal outcomes, advanced age independently increased the odds of death (OR = 1.66, 95% CI 1.3–2.1, *p* < 0.001). Tumor type had a strong effect: relative to breast cancer, melanoma (OR = 7.1, *p* < 0.001), other tumors (OR = 7.9, *p* < 0.001), and NSCLC (OR = 4.5, *p* = 0.002) carried markedly higher risks, while treatment regimen was not an independent predictor (OR = 0.90 for combination vs. monotherapy, *p* = 0.45). CTLA-4 use trended toward higher fatality (OR = 1.5, *p* = 0.09), whereas later calendar period (2017–2024) was protective (OR = 0.16 vs. 2011–2016, *p* < 0.001). A parallel hospitalization model showed male sex (OR = 1.35, *p* = 0.018), younger age (OR = 0.74, *p* = 0.014), CTLA-4 use (OR = 2.9, *p* < 0.001), and non-combination regimens (OR = 0.35 for combination, *p* < 0.001) as independent predictors. Sensitivity analyses restricted to each patient’s first ADR were concordant with the main findings (Supplementary Figure S5).

## 4. Discussion

In this national pharmacovigilance cohort (*n* = 2300), chronological age was not associated with increased odds of reporting a serious ICI-related adverse event or with higher rates of hospitalization; however, older adults (≥70 years) had a substantially higher case-fatality (25.3% vs. 16.0%; adjusted OR 1.66, 95% CI 1.31–2.09). Age was further associated with a shift in toxicity phenotype: older patients experienced proportionally more nervous and immune system events and fewer hepatobiliary and hematological events, whereas the overall frequency of explicitly immune-mediated events remained similar across age groups. These findings suggest that age might primarily influence outcomes and toxicity phenotypes rather than the incidence of adverse events.

Patients aged ≥70 years comprised approximately one-third of the cohort, with the proportion of serious ADRs being essentially identical to those <70 years (84.5% vs. 86.5%). In adjusted analyses, age was not an independent predictor of a report being serious (OR 0.98; 95% CI 0.76–1.27; *p* = 0.89). Taken together, these data indicate that chronological age alone does not increase the incidence of immunotherapy toxicities, consistent with prior reports showing comparable any-grade and high-grade irAEs rates in older adults [21,24,32], including a series of patients ≥ 80 years [25]. However, when toxicity does occur in older populations, the prognosis is notably poorer, with fatality rates among reported irAEs being 1.77-fold higher in patients aged ≥70 compared to younger counterparts, and advanced age persisting as an independent predictor of mortality in multivariable modeling. This suggests that chronological age, rather than elevating the incidence of toxicity, may amplify its clinical consequences. Plausible contributors include biological frailty and reduced physiological reserve (e.g., an immune-mediated pneumonitis or colitis that is survivable at younger ages may be lethal with limited cardiopulmonary or metabolic reserve), as well as comorbidity burden (e.g., underlying interstitial lung disease) that magnifies organ-specific injury [21,22,24,25,32]. Differences in management may also play a role: clinicians may be more conservative with escalation or high-dose corticosteroids in the very old, influenced by concerns about treatment complications or perceived lower survival potential and higher care burden [20,22]. Notably, hospitalization was not more frequent in ≥70 years (slightly lower, non-significant), which could reflect outpatient/hospice management, under-notification of admissions in the elderly, or deterioration preceding admission. Overall, these data align with clinical experience that older adults are more vulnerable to the consequences of severe irAEs despite a similar baseline risk of experiencing them. This increased vulnerability is biologically plausible in the context of immunosenescence, characterized by thymic involution, reduced naïve T-cell output, expansion of autoreactive memory clones, and dysregulated immune homeostasis, which alters how the aging immune system responds to immune activation and may impair recovery from tissue injury [24,26,27,28]. Interestingly, we observed distinct SOC patterns by age: older adults had relatively more immune system and nervous system disorders, whereas younger patients showed higher hepatobiliary and hematologic events. These distributions are consistent with the organ-specific profiles shown in Supplementary Figure S3 and Supplementary Table S2. Prior work also suggests age-linked shifts in irAE spectra, e.g., more dermatologic/rheumatologic toxicities in older adults and more colitis/hepatitis in younger cohorts, although findings have been heterogeneous [22,25,32,33]. Our data are partly concordant: fewer hepatic events in ≥70 years (consistent with a less reactive hepatic immune milieu) and a numerical increase in endocrine events with age, albeit non-significant [9,26,32]. The higher neurologic irAEs signal in the elderly is notable; given their seriousness and relative rarity, this could reflect greater biological susceptibility (frailty of neural/blood–brain barrier repair) or differential recognition/reporting [14,15,20,22,34]. Conversely, younger patients’ excess hematologic events may align with a more vigorous immune repertoire capable of autoimmune cytopenias. Mechanistically, immunosenescence, with dampened effector responses but expansion of autoreactive memory clones, could shift organ vulnerability and clinical expression [22,24,32,33,35]. Clinically, these patterns argue for age-attuned surveillance (e.g., higher suspicion for encephalitis in the elderly vs. hepatitis in younger adults) [25,26]. Importantly, the overall irAE frequency was comparable across ages; the difference lies in the proportions, not presence, of organ-specific toxicities.

In further exploratory, adjusted analyses, combination therapy independently increased the odds of an ADR being classified as *serious* (aOR 1.57), although it did not raise mortality rates within the cohort. Regimens containing CTLA-4 inhibitors were associated with the most unfavorable crude outcomes, including higher rates of fatality and hospitalization, consistent with established toxicity profiles of these agents [14,15,36,37]. Tumor type remained associated with seriousness/fatality (e.g., higher seriousness in renal cell carcinoma (RCC) and breast vs. “other”), likely reflecting regimen mix and case-mix. Sex showed little difference for overall seriousness or death, but phenotype diverged, more endocrine events in women; more respiratory/vascular toxicities and higher hospitalization in men, aligning with prior sex-immune literature [38,39,40]. Polypharmacy and calendar period were not independent drivers of *serious* vs. *non-serious* after adjustment, although 2017–2024 was associated with lower fatality, consistent with the transition to PD-(L)1 agents and maturing irAE management [14,15,36]. Full coefficients and forest plots are provided in the Supplementary Materials.

This analysis is constrained by the well-recognized biases of spontaneous pharmacovigilance data. First, severity bias and under-reporting likely inflate the proportion of serious and fatal cases captured: 86% of reports in our cohort were classified as *serious* and 19% as *fatal*, figures that far exceed trial-based estimates of fatal irAEs (<1%) and large real-world series (0.6%), reflecting selective reporting rather than population risk [36,41,42,43]. Spontaneous systems miss most ADRs (median under-reporting 94%; even serious ADRs 80–85% under-reported), so mild/moderate toxicities are under-ascertained [44,45]. Second, event misclassification and incomplete granularity are probable. The designation “immune-mediated” versus other ADRs relied on reporter judgment/MedDRA coding; classical irAEs may have been filed under organ SOCs (e.g., hepatitis, colitis) or, conversely, non-immune events over-attributed to ICIs. Timing data were sparse and sometimes imprecise (frequent “day-0” entries), precluding robust latency analyses [42,45]. Third, residual confounding cannot be excluded. Indication and regimen (e.g., CTLA-4 exposure) correlate with both toxicity and reporting; multivariable adjustment attenuated but did not remove confounding. The model for *serious* vs. *non-serious* had low explanatory power (pseudo-R^2^ 0.02), suggesting unmeasured determinants (toxicity phenotype, management thresholds, reporter behavior). Given the very low pseudo-R^2^ (0.02), the seriousness model has limited predictive power and should be interpreted as identifying the direction rather than the magnitude of associations. Fourth, missing covariates and unknown denominators limit inference. We lacked performance status, comorbidities, laboratory values, and treatment setting; polypharmacy was an imperfect proxy for comorbidity. The number of ICI-treated patients in Portugal is unknown, so incidence cannot be estimated; findings characterize risk patterns within reported ADRs, not absolute rates. Finally, age was modeled primarily as <70/≥70 (chosen a priori); while consistent with sensitivity analyses, very-old-age effects may be obscured, and differential reporting across subgroups cannot be excluded.

Taken together, these real-world findings suggest that chronological age, per se, should not preclude immunotherapy. Older adults (≥70 y) were no more likely than younger patients to experience an ADR classified as “serious”, yet, once a toxicity occurred, the risk of death was higher in the older group. This pattern supports equitable access to ICIs for fit older people, paired with age-attuned safety nets to mitigate consequence-of-toxicity risk [14,15,20,25,33,34]. The age signal we observed was phenotypic rather than incidence-based: older adults had relatively more nervous-system and immune-system disorders, whereas younger adults showed more hepatobiliary and blood/lymphatic events. Clinically, this warrants a lower threshold in ≥70 y for urgent evaluation and early immunosuppression (e.g., corticosteroids per ESMO/ASCO guidance) when facing encephalopathy, neuropathy, myasthenic syndromes, aseptic meningitis/encephalitis, or multisystem immune activation; for <70 y, maintaining routine liver chemistries and complete blood counts through early cycles remains high yield to detect immune hepatitis and cytopenias at presentation [14,15,20,22]. Regardless of age, new dyspnea, diarrhea, chest pain or severe fatigue should trigger protocolized same-day work-ups. To operationalize vigilance, centers should front-load monitoring in cycles 1–2, the window in which many irAEs declare, by (i) pre-authorizing rapid-access irAEs clinics with same-day labs and imaging; (ii) implementing escalation checklists for older adults (triggers for ED evaluation/admission and step-up to high-dose steroids or second-line agents); and (iii) scheduling 48–72 h follow-up (in person or telemedicine) after any grade ≥ 2 toxicity until improvement. Embedding irAE order sets and steroid-taper templates within the electronic record reduces delays and variation [14,15,20]. Because the hazard in older adults relates to outcome severity rather than event frequency, pre-treatment geriatric assessment should guide monitoring intensity and thresholds for intervention. A brief clinic-feasible bundle (mobility/falls, polypharmacy, cognition, nutrition, social support, comorbidity burden) can stratify physiological reserve and help anticipate toxicities that older patients tolerate poorly (pneumonitis, myocarditis, neuro-irAEs). The development and validation of a pragmatic score to estimate fatal/near-fatal irAEs risk in older adults is a priority [20,22,33,34]. CTLA-4-containing schedules require heightened caution: baseline stool history, early GI input, and a “treat-early” steroid stance for diarrhea/abdominal pain to prevent complicated colitis, the dominant cause of ipilimumab-related deaths in prior reports [14,15,28]. For PD-(L)1 monotherapy, the emphasis is on thyroid/adrenal surveillance and prompt work-up of respiratory or hepatic symptoms. For combination regimens, which in our data increased the odds of being classified as serious but did not increase mortality, proactive education and scheduled toxicity check-ins often keep events from escalating, and higher event frequency need not translate into higher lethality when structured management is in place [14,15,36]. A practical cadence is weekly labs during cycle 1 (ALT/AST/bilirubin, ALP, CBC; TSH/FT4 and morning cortisol if symptomatic), then at each infusion through cycle 4, with earlier repetition for symptoms. In ≥70 y with headache, confusion, gait change or focal deficits, adopt a low threshold for brain MRI and neurology consultation; maintain a low threshold for CT chest and pulse-oximetry trending with any new cough or exertional dyspnea in all ages [14,15,20]. Outcomes improved over time, plausibly reflecting better clinician education and earlier intervention [14,15,20,28]. Continuing this trajectory requires patient-centered education (plain-language action plans; wallet cards/app-based alerts; 24/7 contact pathways) and onboarding of primary-care and emergency teams, since first presentations often occur outside oncology clinics. Given the higher case-fatality in older adults, caregivers should be explicitly enlisted to report early neurologic or constitutional changes.

Our analysis underscores the value, and current limitations, of spontaneous reporting. Enhancing pharmacovigilance for ICIs with structured fields (onset date, CTCAE grade, management, outcome) and encouraging reporting of all grades of suspected irAEs would improve signal quality and refine practice benchmarks [24,46]. Prospective registries and patient-reported tools could better capture mild/moderate events that shape quality of life and adherence. As spontaneous reporting lacks a denominator, true incidence cannot be assessed. In practical terms, clinicians should counsel that most older adults will not suffer fatal toxicity, but the margin for delay is smaller. For borderline-fit patients, regimen choice (e.g., PD-1 alone rather than PD-1 + CTLA-4) can meaningfully reduce risk without foreclosing benefit [14,15,28]. As biomarker research matures (HLA, cytokines, microbiome), integration with geriatric metrics may further personalize monitoring and prophylaxis; until then, phenotype-aware surveillance, rapid escalation pathways, and geriatric-informed care are the highest-yield levers to maximize safety while preserving access to life-prolonging ICIs [20,22,33,34,36].

## 5. Conclusions

In this national pharmacovigilance cohort, chronological age did not increase the odds of experiencing an ICI-related adverse event, supporting the use of ICIs in fit older adults; however, when irAEs occurred in patients ≥70 years, the risk of fatal outcome was higher, underscoring the need for earlier recognition and lower thresholds for escalation in this group. Toxicity phenotype varied by age (more nervous/immune-system disorders in older adults; more hepatobiliary/hematologic events in younger adults), by sex (more endocrine events in women; more respiratory/vascular events in men), and, most strongly, by regimen: CTLA-4-containing schedules showed the severest profiles, whereas PD-1/PD-L1 monotherapy (e.g., in NSCLC) showed a better safety outcome. Combination therapy increased the volume of reported toxicity but did not increase mortality, consistent with effective monitoring and management. Temporal trends suggest improving safety with wider PD-1/PD-L1 adoption and greater clinician experience. Taken together, these findings highlight the complex interplay between age, immune function, and the expression of irAEs, reflecting the biological heterogeneity introduced by age-related immune remodeling. Although *immunosenescence* may not increase the likelihood of developing an irAE, it may influence how toxicities manifest and how well patients recover once severe inflammation occurs. As far as we are aware, this is also the first national real-world evaluation of ICI safety in Portugal, offering a comprehensive perspective on how these therapies perform outside trial settings. These findings, while influenced by the severity bias of spontaneous reporting, provide actionable signals for practice: maintaining access to ICIs for older adults, implementing front-loaded monitoring and rapid-response pathways, especially for CTLA-4 regimens, and tailoring surveillance by patient phenotype. Reporting of irAEs should be actively encouraged. Prospective registry linkages and development of pragmatic geriatric risk tools should be prioritized to refine risk stratification and further reduce fatal irAEs.

## Figures and Tables

**Table 1:** Baseline Characteristics of ICI-Related Adverse Drug Reaction Cases by Age Group.

Variable		All Events (*n* = 2300)	Age < 70 Years (*n* = 1525)	Age ≥ 70 Years (*n* = 775)
**Age (years)**				
	median	65	59	76
	min-max	20–93	20–69	70–93
**Sex—*n* (%)**				
	Male	1444 (62.8)	888 (58.2)	556 (71.7)
	Female	856 (37.2)	637 (41.8)	219 (28.3)
**Tumor type—*n* (%)**				
	Melanoma	339 (14.7)	233 (15.3)	106 (13.7)
	NSCLC	719 (31.3)	476 (31.2)	243 (31.4)
	RCC	190 (8.3)	133 (8.7)	57 (7.4)
	Breast	187 (8.1)	172 (11.3)	15 (1.9)
	Others	865 (37.6)	511 (33.5)	354 (45.7)
**ICI class—*n* (%)**				
	PD-1	1783 (77.5)	1211 (79.4)	572 (73.8)
	PD-L1	348 (15.1)	187 (12.3)	161 (20.8)
	CTLA-4	112 (4.9)	89 (5.8)	23 (3.0)
	PD-1 + CTLA-4	51 (2.2)	35 (2.3)	16 (2.1)
	PD-L1 + CTLA-4	5 (0.2)	3 (0.2)	2 (0.3)
	iPD-L1 + iCD73	1 (0.0)	0 (0)	1 (0.1)
**Treatment protocol—*n* (%)**				
	Monotherapy	1676 (72.9)	1020 (66.9)	656 (84.6)
	Combination *	624 (27.1)	505 (33.1)	119 (15.4)
**Concomitant medications—*n* (%)**				
	median	1	1	1
	0–2	1770 (77.0)	1226 (80.4)	544 (70.2)
	3–5	272 (11.8)	184 (12.1)	88 (11.4)
	>5	258 (11.2)	115 (7.5)	143 (18.5)
**Calendar period—*n* (%)**				
	2011–2016	167 (7.3)	104 (6.8)	63 (8.1)
	2017–2024	2133 (92.7)	1421 (93.2)	712 (91.9)

Baseline characteristics of immune checkpoint inhibitor (ICI)-related adverse drug reaction (ADR) cases, presented overall and stratified by age group (<70 years vs. ≥70 years). Variables include age distribution, sex, tumor type, ICI class, treatment protocol (monotherapy vs. combination therapy), number of concomitant medications, and calendar period of reporting. Data are shown as number (percentage) or median (range), based on 2300 ADR cases (1525 in patients <70 years and 775 in patients ≥70 years). Abbreviations: n, number; NSCLC, non-small cell lung cancer; RCC, renal cell carcinoma; ICI, immune checkpoint inhibitor; PD-1, programmed cell death protein 1; PD-L1, programmed death-ligand 1; CTLA-4, cytotoxic T-lymphocyte–associated protein 4; iPD-L1, inhibitor of programmed death-ligand 1; iCD73, inhibitor of CD73. * combinations included dual checkpoint blockade or ICI plus chemotherapy.

**Table 2:** Distribution of adverse events by System Organ Class in patients < 70 and ≥70 years treated with immune checkpoint inhibitors.

System Organ Class	<70 Years	≥70 Years	*Sig. p*-Value
Blood and lymphatic disorders	7.0%	3.8%	*0.017*
Cardiac disorders	2.9%	2.9%	
Ear and labyrinth disorders	0.1%	0.1%	
Endocrine disorders	8.7%	11.4%	
Eye disorders	0.8%	0.9%	
Gastrointestinal disorders	8.3%	8.9%	
General disorders and administration-site conditions	12.0%	10.7%	
Hepatobiliary disorders	7.2%	3.8%	*0.004*
Immune system disorders	6.6%	9.3%	*0.039*
Infections and infestations	4.1%	4.2%	
Injury, poisoning and procedural complications	1.1%	0.8%	
Investigations (lab/test abnormalities)	2.6%	2.6%	
Metabolism and nutrition disorders	3.9%	4.4%	
Musculoskeletal and connective tissue disorders	5.3%	5.7%	
Nervous system disorders	5.1%	8.7%	*0.028*
Product issues	0.3%	0.1%	
Psychiatric disorders	0.9%	0.8%	
Renal and urinary disorders	5.6%	4.6%	
Reproductive system and breast disorders	0.4%	0.0%	
Respiratory, thoracic and mediastinal disorders	7.4%	6.8%	
Skin and subcutaneous disorders	8.2%	9.8%	
Vascular disorders	1.3%	1.7%	

Heatmap showing the proportional distribution of adverse drug reactions (ADRs) by MedDRA System Organ Class (SOC) among patients aged <70 years and ≥70 years treated with immune checkpoint inhibitors. Each cell represents the percentage of total ADRs for that age group falling within the corresponding SOC category. Background color intensity reflects the relative proportion of adverse drug reactions within each System Organ Class, with darker shading indicating higher percentages. Exact values and statistical comparisons are provided in the Supplementary Materials Tables S3 and S4.

## Data Availability

The data used in this study were obtained from the Portuguese National Pharmacovigilance System (INFARMED). Due to legal and GDPR restrictions, individual case safety reports cannot be publicly shared. Aggregated, de-identified data supporting the findings of this study are available from the corresponding author upon reasonable request and with permission from INFARMED.

## Supplementary Materials

The following supporting information can be downloaded at: https://www.mdpi.com/article/10.3390/cancers18010076/s1, Table S1: Seriousness classification, immune-related labeling, hospitalization, and CTCAE-equivalent severity grades of immune checkpoint inhibitor-related adverse drug reactions (ADRs), stratified by age group (<70 vs. ≥70 years); Figure S1: Distribution of CTCAE-equivalent grades of immune checkpoint inhibitor–related ADRs in patients < 70 and ≥70 years; Figure S2: Radar plot of organ-system involvement (MedDRA System Organ Classes) in immune checkpoint inhibitor-related ADRs for patients < 70 years, ≥70 years, and the overall cohort; Figure S3: Age-associated shifts in MedDRA organ-system involvement in immune checkpoint inhibitor-related ADRs, comparing patients < 70 versus ≥70 years; Table S2: Most frequently reported adverse events (top MedDRA Preferred Terms), most frequent immune-related events, and leading MedDRA System Organ Classes, stratified by age group (<70 vs. ≥70 years); Figure S4: Age-stratified graphical distributions of the most common adverse events, most common immune-related events, and major MedDRA System Organ Classes; Table S3: Sex-specific patterns of immune checkpoint inhibitor-related ADRs, including seriousness, fatality, hospitalization, immune-related classification, organ-system distribution, and median time-to-onset; Table S4: Stratified safety outcomes by treatment regimen (monotherapy vs. combination), CTLA-4 exposure, degree of polypharmacy, calendar period, and tumor type; Figure S5: Sensitivity analyses restricted to the first ADR per patient (patient-level dataset), demonstrating robustness of the primary associations.
