# Supplementary material for: Age and Clinical Outcomes of Immune Checkpoint Inhibitor Toxicities in Portugal: A Decade of Pharmacovigilance
Source: Cancers (Basel). 2025 Dec 25;18(1):76. doi: 10.3390/cancers18010076 (PMC12784953; doi:10.3390/cancers18010076)
Supplement: Supplementary file 1 [file cancers-18-00076-s001.zip › cancers-4058653-supplementary.pdf]

Supplementary Materials

The following supporting information can be downloaded at: (link inserted by journal).

All supplementary analyses were conducted on the same curated pharmacovigilance dataset described in the Methods.

Index:

- **Table S1.** Seriousness classification, immune-related labeling, hospitalization, and CTCAE-equivalent severity grades of ICI-related adverse drug reactions (ADRs), stratified by age group (<70 vs ≥70 years).
- **Figure S1.** Distribution of CTCAE-equivalent grades of ICI-related ADRs in patients <70 and ≥70 years.
- **Figure S2.** Radar plot of organ-system involvement (MedDRA System Organ Classes) in ICI-related ADRs for patients <70 years, ≥70 years, and the overall cohort.
- **Figure S3.** Age-associated shifts in MedDRA organ-system involvement in ICI-related ADRs, comparing patients <70 versus ≥70 years.
- **Table S2.** Most frequently reported adverse events (top MedDRA Preferred Terms), most frequent immune-related events, and leading MedDRA System Organ Classes, stratified by age group (<70 vs ≥70 years).
- **Figure S4.** Age-stratified graphical distributions of the most common adverse events, most common immune-related events, and major System Organ Classes.
- **Table S3.** Sex-specific patterns of ICI-related ADRs, including seriousness, fatality, hospitalization, immune-related classification, organ-system distribution, and median time-to-onset.
- **Table S4.** Stratified safety outcomes by treatment regimen (monotherapy vs combination), CTLA-4 exposure, degree of polypharmacy, calendar period, and tumor type.
- **Figure S5.** Sensitivity analyses restricted to the first ADR per patient (patient-level dataset), demonstrating robustness of primary associations.

Table S1. Seriousness, immune-related classification, hospitalization, and CTCAE-equivalent grading of ICI-related adverse drug reactions, stratified by age (<70 vs. ≥70 years). Summary table reporting seriousness (regulatory definition), immune-related labeling, hospitalization rates, and CTCAE-equivalent severity grades across age groups.

**Variable**		**All e2300.**	**Age <70 years (n = 1525)**	**Age ≥70 years (n = 775)**
Seriousness of events – n (%)	Serious	1974 (85.8)	1319 (86.5)	655 (84.5)
	Non-serious	326 (14.2)	206 (13.5)	120 (15.5)
Immune-related events - n(%)		207(9%)	133(8.7)	74(9.5)
Hospitalizations - n(%)		412(17.9)	282(18.5)	130(16.8)
CTCAE grade (n,%)	G1—Non-serious	326 (14.2)	206 (13.5)	120 (15.5)
	G2—Medically important	1234 (53.7)	869 (57.0)	365 (47.1)
	G3—Severe/medically significant	145 (6.2)	103 (6.8)	42 (5.4)
	G4—Life-threatening	155 (6.7)	103 (6.8)	52 (6.7)
	G5—Death	440 (19.1)	244 (16.0)	196 (25.3)

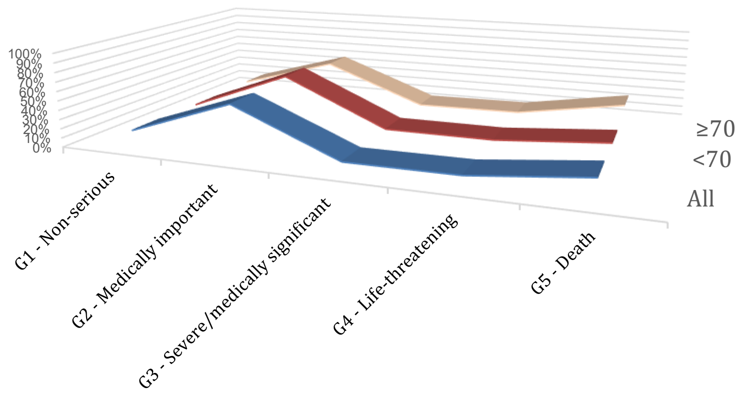

	G1 - Non-serious	G2 - Medically important	G3 - Severe/medically significant	G4 - Life-threatening	G5 - Death
All	14.2%	53.7%	6.3%	6.7%	19.1%
<70	13.5%	57.0%	6.8%	6.8%	16.0%
≥70	15.9%	48.3%	5.6%	6.9%	26.0%

All <70 ≥70

Figure S1. Distribution of CTCAE-equivalent grades of ICI-related adverse events by age group (<70 vs. ≥70 years). Visual representation of grade distribution showing a higher proportion of Grade 5 (fatal) events in older adults.

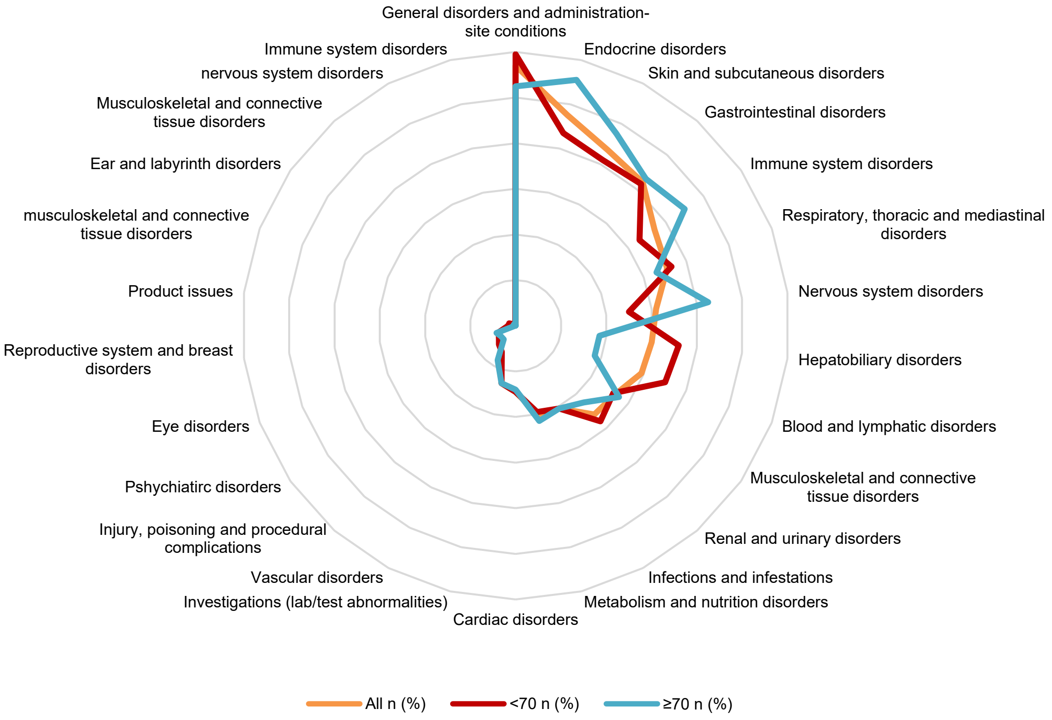

Figure S2. Radar plot of organ system involvement in ICI-related adverse events across MedDRA SOCs by age group (<70, ≥70, overall). Radar chart highlighting age-related redistribution of toxicity phenotypes.

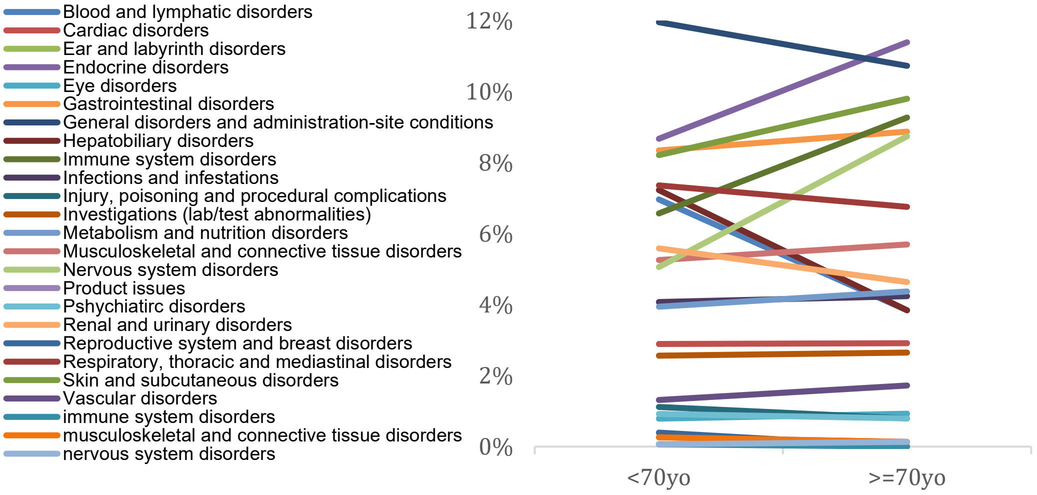

Figure S3. Age-related shifts in organ system involvement of ICI-related adverse events (MedDRA SOCs). Line plot comparing SOC frequencies between age groups, aligned with regression-derived associations.

Table S2. Most frequently reported adverse events (top MedDRA PTs), immune-related events, and organ-class involvement stratified by age (<70 vs. ≥70 years). Includes: Top 10 all-ADR PTs; Top 10 immune-related PTs; Top SOCs. All shown separately for both age strata.

Variable	All		<70 years		≥70 years	
Top 10 reported adverse events (MedDRA PT) - n(%)						
	Asthenia	63 (2.7)	Asthenia	49(3.2)	Hypothyroidism	20(2.6)
	Hypothyroidism	61(2.7)	Hypothyroidism	41(2.7)	Decreased appetite	19(2.5)
	Pneumonitis	53(2.3)	Pneumonitis	36(2.4)	Pneumonitis	17(2.2)
	Diarrhea	49(2.1)	Diarrhea	32(2.1)	Diarrhea	17(2.2)
	Decreased appetite	37(1.6)	Anemia	26(1.7)	Pruritus	15(1.9)
	Arthralgia	32(1.4)	Drug-induced liver injury	26(1.7)	Asthenia	14(1.8)
	Anemia	32(1.4)	Arthralgia	23(1.5)	Diabetic ketoacidosis	14(1.8)
	Pyrexia	31(1.3)	Pyrexia	22(1.4)	Colitis	12(1.5)
	Colitis	30(1.3)	Hepatotoxicity	19(1.2)	Respiratory tract infection	10(1.3)
	Pruritus	28(1.2)	Rash	19(1.2)	Arthralgia	9(1.2)
Top 10 reported immune-related adverse events (MedDRA PT) - n(%)						
	Pneumonitis	53(25.6)	Pneumonitis	36(27.1)	Pneumonitis	17(23)
	Immune-mediated lung disease	22(10.6)	Immune-mediated lung disease	14(10.5)	Immune-mediated hypothyroidism	9(12.2)
	Immune-mediated hypothyroidism	22(10.6)	Immune-mediated hypothyroidism	13(9.8)	Immune-mediated lung disease	8(10.8)
	Immune-mediated hepatitis	16(7.7)	Thyroiditis	10(7.5)	Immune-mediated hepatitis	6(8.1)
	Immune-mediated enterocolitis	13(6.3)	Immune-mediated hepatitis	10(7.5)	Immune-mediated enterocolitis	5(6.8)
	Immune-mediated hypophysitis	12(5.8)	Immune-mediated enterocolitis	8(6)	Immune-mediated hypophysitis	4(5.4)
	Thyroiditis	10(4.8)	Immune-mediated hypophysitis	8(6)	Vitiligo	3(4.1)
	Vitiligo	8(3.9)	Vitiligo	5(3.8)	Immune-mediated myocarditis	3(4.1)
	Immune-mediated thyroiditis	6(2.9)	Immune-mediated thyroiditis	4(3)	Immune-mediated dermatitis	2(2.7)
	Immune-mediated arthritis	5(2.4)	Immune-mediated gastritis	4(3)	Immune-mediated myasthenia gravis	2(2.7)
Top organ-class system involvement (MedDRA SOC) - n(%)						
	General disorders and administration-site conditions	263(11.4)	General disorders and administration-site conditions	182(11.9)	Endocrine disorders	86(11.1)
	Endocrine disorders	218(9.5)	Endocrine disorders	132(8.7)	General disorders and administration-site conditions	81(10.5)
	Skin and subcutaneous disorders	199(8.7)	Gastrointestinal disorders	127(8.3)	Skin and subcutaneous disorders	74(9.5)
	Gastrointestinal disorders	194(8.4)	Skin and subcutaneous disorders	125(8.2)	Immune system disorders	70(9)
	Immune system disorders	170(7.4)	Respiratory, thoracic and mediastinal disorders	112(7.3)	Gastrointestinal disorders	67(8.6)
	Respiratory, thoracic and mediastinal disorders	163(7.1)	Hepatobiliary disorders	110(7.2)	Nervous system disorders	66(8.5)

Nervous system disorders	143(6.2)	Blood and lymphatic disorders	106(7)	Respiratory, thoracic and mediastinal disorders	51(6.6)
Hepatobiliary disorders	139(6)	Immune system disorders	100(6.6)	Musculoskeletal and connective tissue disorders	43(5.5)
Blood and lymphatic disorders	135(5.9)	Renal and urinary disorders	85(5.6)	Renal and urinary disorders	35(4.5)
Musculoskeletal and connective tissue disorders	123(5.3)	Musculoskeletal and connective tissue disorders	80(5.2)	Metabolism and nutrition disorders	33(4.3)

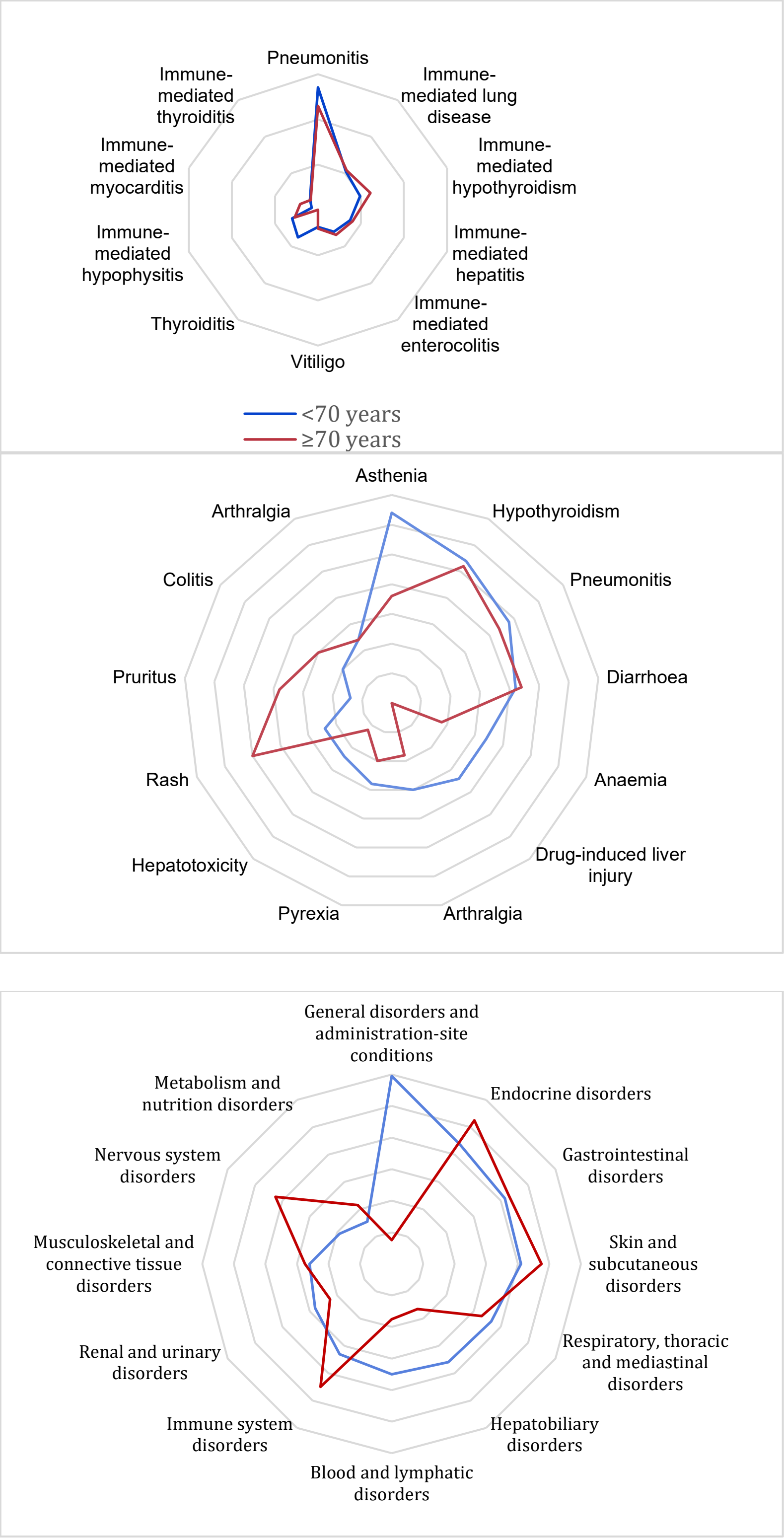

Figure S4. Age-stratified patterns of the most common adverse events, immune-related events, and organ system involvement. Three-panel graphical summary (AEs, irAEs, SOCs) corresponding to Table S2.

Table S3. Sex-specific patterns of ICI-related adverse events, including seriousness, fatality, hospitalization, immune-related labeling, and organ-system distribution.

Variable	Male (n,%)	Female (n,%)	p-value
Seriousness of events			
Serious events	1241 (85.9)	733 (85.6)	0,885
Non-serious events	203 (14.1)	123 (14.4)	
Fatal outcomes	286 (19.8)	154 (18.0)	0,31
Hospitalizations	287 (19.9)	125 (14.6)	0,002
Specific organ system categories			
Immune-related events	141 (9.8)	66 (7.7)	0,112
Blood and lymphatic disorders	64 (4.4)	71 (8.3)	0
Endocrine disorders	113 (7.8)	105 (12.3)	0,001
Vascular disorders	28 (1.9)	5 (0.6)	0,014
Respiratory, thoracic and mediastinal disorders	116 (8.0)	47 (5.5)	0,027
Event onset			
Time-to-onset (median days)	0.0 (IQR 0.0-89.75)	0.0 (IQR 0.0-0.0)	0,054

Includes significance testing for each category and median time-to-onset by sex.

Table S4. Stratified safety outcomes by treatment regimen (monotherapy vs. combination), CTLA-4 exposure, polypharmacy level, calendar period, and tumor type.

Variable	Total	Immune events	Non-serious	Serious	Hospitalizations	Fatal
Regimen (mono vs combo)						
Monotherapy	1676	169 (10.1)	270 (16.1)	1406 (83.9)	365 (21.8)	355 (21.2)
Combination	624	38 (6.1)	56 (9.0)	568 (91.0)	47 (7.5)	85 (13.6)
Regemin (CTLA-4)						
Non CTLA-4	2132	183 (8.6)	309 (14.5)	1823 (85.5)	339 (15.9)	372 (17.4)
CTLA-4 containing	168	24 (14.3)	17 (10.1)	151 (89.9)	73 (43.5)	68 (40.5)
Polypharmacy						
≤2	1770	177 (10.0)	238 (13.4)	1532 (86.6)	296 (16.7)	351 (19.8)
3-5	272	17 (6.3)	38 (14.0)	234 (86.0)	78 (28.7)	30 (11.0)
>5	258	13 (5.0)	50 (19.4)	208 (80.6)	38 (14.7)	59 (22.9)
Calendar period						
2011–2016	172	6 (3.6)	15 (8.7)	157 (91.3)	53 (30.8)	101 (58.7)
2017–2024	1599	111 (7.2)	219 (13.7)	1380 (86.3)	275 (17.2)	228 (14.3)
Tumor groups						
NSCLC	848	721 (85.0)	127 (15.0)	182 (21.5)	125 (14.7)	52 (6.1)
Melanoma	719	587 (81.6)	132 (18.4)	107 (14.9)	153 (21.3)	93 (12.9)
RCC	356	314 (88.2)	42 (11.8)	106 (29.8)	130 (36.5)	36 (10.1)
Breast	190	178 (93.7)	12 (6.3)	40 (21.1)	4 (2.1)	12 (6.3)
Other	187	174 (93.0)	13 (7.0)	5 (2.7)	0 (0.0)	14 (7.5)

Reports seriousness, immune-related classification, hospitalization, and fatal outcomes.

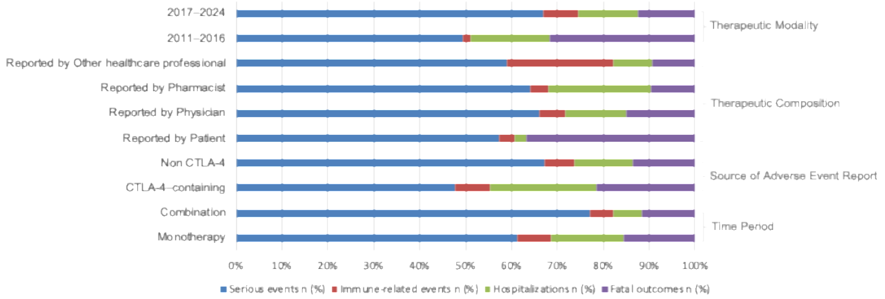

Figure S5. Sensitivity analyses using first ADR per patient (patient-level dataset). Graphs visualizing the robustness of associations after de-duplication at the patient level.
